# Clinical and genetic characteristics of children with COX20-associated mitochondrial disorder: case report and literature review

**DOI:** 10.1186/s12920-023-01513-y

**Published:** 2023-04-24

**Authors:** Liqing Chen, Yan Liu

**Affiliations:** Department of Pediatrics, Tongji Hospital, Tongji Medical College, Huazhong University of Science and Technology, Wuhan, China

**Keywords:** COX20, ataxia, Sensory neuropathy, Visual impairment, Case report

## Abstract

**Background:**

The deficiency of cytochrome c oxidase 20 is a rare autosomal recessive mitochondrial disorder characterized by ataxia, dysarthria, dystonia and sensory neuropathy.

**Case presentation:**

In this study, we describe a patient from a non-consanguineous family exhibiting developmental delay, ataxia, hypotonia, dysarthria, strabismus, visual impairment and areflexia. An examination of nerve conduction showed a normal result at first but revealed axonal sensory neuropathy later. This situation has not been reported in any literatures. The whole-exome sequencing analysis revealed that the patient harbored compound heterozygous mutations (c.41 A > G and c.259G > T) of the COX20 gene. By literature review, 5 patients carried the same compound heterozygous mutations.

**Conclusion:**

COX20 might be considered as a potential gene for the early-onset ataxia and the axonal sensory neuropathy. Our patient exhibited strabismus and visual impairment, which expands the clinical presentation of COX20 related mitochondrial disorders caused by the compound heterozygous variants (c.41 A > G and c.259G > T). However, a clear genotype/phenotype correlation has not yet been established. Additional researches and cases are needed to further confirm the correlation.

**Supplementary Information:**

The online version contains supplementary material available at 10.1186/s12920-023-01513-y.

## Background

Cytochrome c oxidase (COX) / Complex IV (CIV) is a mitochondrial inner membrane multi-subunit enzyme encoded by both mitochondrial DNA and nuclear genome. The mitochondrial DNA encodes the three subunits (COX1, COX2, and COX3) that compose the catalytic core of the enzyme, while nuclear DNA encodes the remaining subunits [1]. As one of the nuclear genes, the COX20 gene encodes a protein which is an essential assembly factor of mitochondrial CIV. The COX20 protein functions as a chaperone that stabilizes the newly synthesized catalytic core subunit COX2 [1]. It also interacts with the COX2-specific metallochaperones SCO1 and SCO2 in the maturation of the COX2 redox center [2]. In the absence of COX20, COX2 is inefficiently integrated into Complex IV subassemblies, resulting in a decrease in the enzyme activity and protein level of Complex IV [5]. Complex IV is the terminal oxidase of the mitochondrial respiratory chain, with its deficiency being a primary cause of oxidative phosphorylation (OXPHOS) disorders [3]. Previous studies have confirmed that the variants of the COX20 gene can lead to decrease of the protein level of COX20, the impairment of the assembly of mitochondrial Complex IV and the deficiency of the function of OXPHOS [4]. To date, a total of 29 patients with COX20 deficiency have been reported [4–12]. The most frequent clinical features of COX20-associated mitochondrial disorder includes sensory neuropathy, ataxia, dysarthria, hypotonia, dystonia, and ophthalmoplegia [12].

Here we report a Chinese child with COX20 deficiency, who manifested developmental delay, ataxia, hypotonia, dysarthria, strabismus and visual impairment. By literature review, the clinical phenotype and genetic characteristics were analyzed.

## Case presentation

The index patient was a 5-year-old boy from a non-consanguineous family, without a history of neurologic diseases. He was born at term without any neonatal problems. His elder sister was healthy. He was able to walk at the age of sixteen months. Since he began to walk, he has displayed a waddling gait and a propensity to fall. He began to speak at the age of one, but in a slurred way. At the age of 4.5, the patient underwent magnetic resonance imaging (MRI) and nerve conduction tests, but all the results were normal. However a neurological examination revealed hypotonia, the absence of tendon reflexes, and a positive finger-nose test. At the age of 5, he suffered from strabismus and visual impairment. He was admitted to our hospital at the age of 5. He still had a slurred speech and abnormal gait, but his cognition was normal. A neurological examination performed on him, revealed hypotonia, Romberg sign, and the absence of deep tendon reflexes. A finger-nose test performed on him showed a positive result. No foot and joint deformities were observed. Sensory examination showed a normal result. The results of complete blood counting, homocysteine, lactate, ammonia, pyruvic acid, urine organic acids, and plasma amino acids were in the normal range. The liver and renal function tests and the cerebrospinal fluid (CSF) analysis performed on him all showed an unremarkable result. An MRI of his brain and spine showed a normal result. However, the examination of nerve conduction performed on him again revealed severe axonal sensory neuropathy. Although the compound muscle action potential (CMAP) amplitude and motor conduction velocity (MCV) of the bilateral tibial nerve and common peroneal nerve were normal, the sensory nerve action potential (SNAP) amplitude of the bilateral sural nerve and superficial peroneal nerve could not be elicited. His right eye’s visual acuity was 0.4 and his left eye’s 0.6, while the fundus oculi examination and the orbital MRI test showed a normal result.

**Fig. 1:**
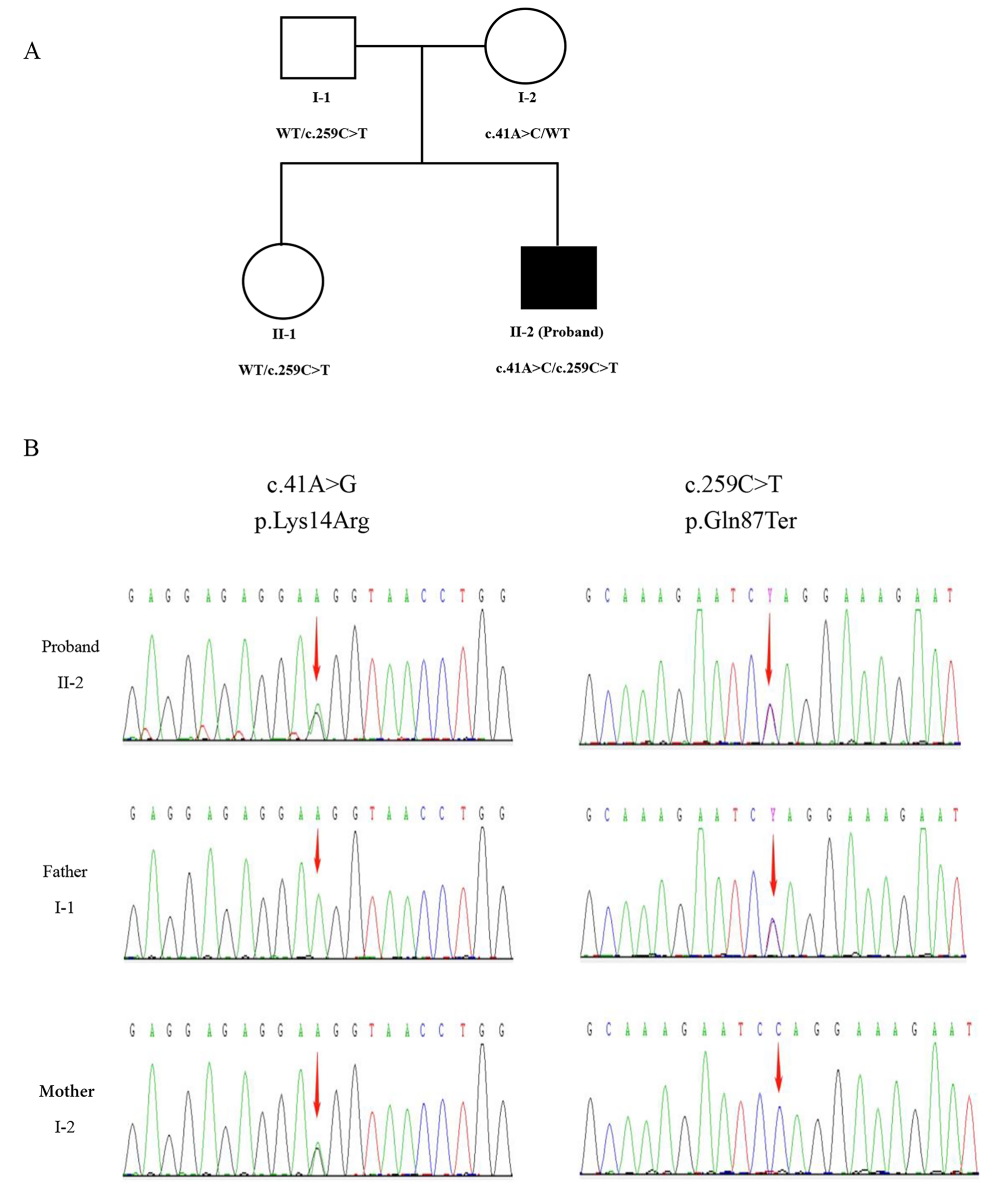
(A) Pedigree of the COX20 family. (B) Chromatographs of the COX20 variants of all family members

The compound heterozygous mutations (c.41 A > G and c.259G > T) were identified in the COX20 gene by whole-exome sequencing (WES), while his unaffected mother carried c.41 A > C and his unaffected father carried c.259G > T in the heterozygous form. (Fig. 1) Located within the donor splice of exon 1, the missense c.41 A > G (p.Lys14Arg) mutation may result in a 20 bp deletion. As has been proven, this variant results in aberrant splicing and the premature termination codon p.Gly8Valfs*2, which further leads to nonsense-mediated mRNA degradation by producing a shorter and more unstable transcript [4, 11]. Located within the exon 4, the nonsense c.259 C > T (p.Gln87Ter) mutation results in premature termination of the coding protein in exon4, and the length of the missing coding protein exceeds 10% of the normal coding protein, which impairs the function of the coding protein product. The c.259 C > T (p.Gln87Ter) mutation has been reported by Dong and Ban et al. [4, 12].

L-carnitine and coenzyme Q10 were administered to the patient, but the medication had no effect. Now he is 6 years old, he falls more frequently than before.

## Literature review of patients with COX20 gene mutation (c.41 A > G and c.259 G > T)

To date, a total of 10 mutations in the COX20 gene have been identified all over the world. Our patient shared the identical COX20 gene mutations (c.41 A > G and c.259G > T) with four other patients from the literature [4, 12]. The clinical features and electrophysiological findings of the patients with these compound heterozygous mutations were summarized in Table 1. Of them, four were males and one was a female. The median age of their onset was between 1 and 2 years; and their initial symptom was difficulty in walking. All patients came from China and they all had ataxia and areflexia. Of them, four showed dysarthria, three showed bilateral foot drop, three showed muscle weakness, and two showed delayed motor milestones. Nerve conduction studies (NCS) were performed on all patients. Four exhibited no residual SNAP while one exhibited an extremely low SNAP. Two exhibited normal MCV and CMAP, indicating pure axonal sensory neuropathy. Two exhibited a normal or decreased MCV in addition to a low CMAP amplitude, indicating mixed sensorimotor axonal neuropathy. One exhibited a reduced MCV and a normal CMAP amplitude indicating axonal sensory and demyelinating motor neuropathy. One’s brain MRI showed thalamus T2 high single while the others’ turned out to be normal. It is worth noting that only this patient in our study exhibited strabismus and visual impairment.

**Table 1:** Summary of patients with compound heterozygous mutations (c.41 A > G and c.259 C > T)

Pt/sex/age of onset	Clinical features							MRI	NCS	Outcome	Ref
	Ataxia	Dysarthria	Dystonia	Development delay	Bilateral foot drop	Areflexia	Other				
1/M/ 1.5y	+	+	-	+	+	+	Mild muscle weakness of distal lower limbs	-	decreased MCV, normal CMAP amplitude; SNAP extremely low	Gait disturbance (11y)	Dong HL et al.
2/F/ 1.5y	+	-	-	-	-	+	Muscle weakness	Thalamus-T2 high single	MCV, CMAP normal; SNAP not elicited	Gait disturbance (9y)	Rui B et al.
3/M/ 2y	+	+	-	-	+	+	Muscle weakness	-	decreased MCV, low CMAP amplitude; SNAP: not elicited	Nonambulatory (10y)	Rui B et al.
4/M/ 1y	+	+	-	-	+	+	-	-	normal MCV, low CMAP amplitude; SNAP: not elicited	Foot drop and difficult to walk(3.5y)	Rui B et al.
5/M/ 1.4y	+	+	-	+	-	+	strabismus and visual failure	-	MCV, CMAP normal; SNAP: not elicited	Gait disturbance (6y)	This study

Pt: patient; MRI: magnetic resonance imaging; NCS: nerve conduction studies; M: male; F: female; MCV: motor conduction velocity; CMAP: compound muscle action potential; SNAP: sensory nerve action potential

## Discussion and conclusions

In the past decade, mitochondrial genes and nuclear genes associated with mitochondrial structure and function have been identified. As one of the nuclear genes, the COX20 gene can influence mitochondrial oxidative phosphorylation (OXPHOS) by encoding a complex IV assembly factor. The decrease in stability and the inability to construct the mature complex IV can result in severe early-onset neuromuscular disorders in humans. The first case of COX20 deficiency was reported by Szklarczyk et al. It was an autosomal recessive form of infanthood-onset mitochondrial disorder which is accompanied by cerebellar ataxia, hypotonia, and developmental delay [5]. Subsequent studies have broadened the phenotypic spectrum. According to the age of onset and the clinical and molecular genetic features, Ban et al. proposed three clinical sub-groups: Infanthood-onset hypotonia without neuropathy, early-childhood-onset axonal neuropathy, later-childhood-onset neuropathy/ dystonia ataxia overlapping syndrome [12].

We reported a compound heterozygous mutation (c.41 A > G, c.259 C > T) of COX20 gene in a Chinese boy with early-childhood-onset axonal neuropathy. The patients in this phenotypical subgroup showed a median onset age of 3 years, severe axonal neuropathy and an earlier onset of neuropathy but no dystonia. Our patient learned to walk in 16 months but his gait remained unsteady. It reveals that his onset age was approximately one year old. However, at the age of 4.5, the examination of nerve conduction showed a normal result. At the age of 5, nerve conduction re-examination revealed severe axonal sensory neuropathy. This situation has not been reported in previous publications. In our opinion, sensory neuropathy, not an initial symptom, may exhibit a threshold effect. At the age of 5, our patient presented strabismus and visual impairment. Only one publication described visual impairment in two siblings associated with the COX20 gene, who carried the compound heterozygous mutations (c.41 A > G, c.222G > T) [11]. Multiple variants have been identified as the cause of visual impairment in mitochondrial diseases. Due to the high energy requirements for electrical transmission, the unmyelinated prelaminar and laminar parts of the optic nerve are rich in mitochondria [13]. The optic nerve is susceptible to minor changes in mitochondrial activity. In our opinion, visual impairment could be a phenotype of patients with COX20 deficiency. The patient develops new symptoms as he grows up, and the original symptoms have a tendency to deteriorate. According to previous research, COX20 deficiency slowed cell growth and impaired cell viability under oxidative stress [4]. Moreover, mitochondria exhibit threshold effects. This may explain the phenomenon.

Additionally, we summarized other four patients from the literatures with the same compound heterozygous mutations (c.41 A > C, c.259 C > T). They were all in the early-childhood-onset axonal neuropathy subgroup, with a median onset age between 1 and 2 years. Ataxia was the initial symptom in each patient. No cognitive impairment or dystonia was identified. Ataxia, dysarthria, bilateral foot drop, areflexia and mild muscle weakness were the most common phenotypes. Only our patient exhibited strabismus and visual impairment, which expands the clinical presentation of COX20 related mitochondrial disorders caused by the compound heterozygous variants (c.41 A > G and c.259G > T). NCS of all patients revealed severe axonal sensory neuropathy. No patient presented cerebral atrophy or spinal atrophy on MRI. The clinical features of all patients were similar, but not identical. This may demonstrate that patients’ phenotypes are influenced not just by their genotypes but also by their families and the environment. Confirming which components contribute to the determination of phenotypes requires additional researches and cases.

Although 29 patients have been reported to date, only 10 pathogenic COX20 gene mutations have been identified. Among the 29 patients, 3 Turkish patients from 2 unrelated families were all reported to carry the same pathogenic homozygous mutation (c.154 A > C, pT52p) [5, 8]; one Turkish patient carried a homozygous mutation (c.190 A > C, pT4P) [9]; 6 American patients from 4 unrelated families all carried the same compound heterozygous mutations (c.41 A > G, c.157 + 3G > C) [7, 10], and the other 19 patients from China all carried the frequently observed c.41 A > G (p.Lys14Arg) mutation, which occurred as a homozygous and compound heterozygous variant. It appears that distinct hot spot mutations of COX20 gene were presented in diverse regions and the population at risk is Chinese. In addition, the c.41 A > G variation was identified in 25 patients excluding four Turkish patients. Located in the donor splice of exon 1, the missense variant c.41 A > G has been demonstrated to cause aberrant splicing and a premature termination codon [4, 14]. Dong et al. postulated an idea that the c.41 A > G was a founder variant in Chinese population [4]. It should be noted that while missense, splice site and nonsense variants in the COX20 gene have been reported, there is currently no clear genotype/phenotype correlation established. Previous studies have observed almost no COX20 protein expression in the patients with the homozygous c.41 A > G (p.Lys14Arg) variant [4]. However, patients carrying this homozygous variation do not exhibit the severest clinical symptoms. It appears that the expression level of COX20 has little association with phenotypic differences.

In summary, mutations in the COX20 gene should be considered in cases with early-onset ataxia and sensory neuropathy. NCS of all patients with COX20 deficiency revealed no residual SNAP or extremely low SNAP, which can substantially aid in the diagnosis of COX20-related mitochondrial disorder. It is worth noting that NCS of our patient was normal at first. Follow-up is very important. However, a clear genotype/phenotype correlation has not yet been established. Additional researches and cases are needed to further confirm the correlation.

## Electronic supplementary material

Below is the link to the electronic supplementary material.

Supplementary Material 1

## Data Availability

The raw datasets generated and analyzed during the current study are not publicly available in order to protect participant confidentiality. The datasets obtained during the current study are available from the corresponding author if the requirements are reasonable.

## Abbreviations

COX: Cytochrome c oxidase 20
CIV: Complex IV
OXPHOS: Oxidative phosphorylation
MRI: Magnetic resonance imaging
CSF: Cerebrospinal fluid
CMAP: Compound muscle action potential
MCV: Motor conduction velocity
SNAP: Sensory nerve action potential
WGS: Whole-exome sequencing
NCS: Nerve conduction studies.
